# Menstrual hygiene management practice among adolescent girls: an urban–rural comparative study in Rajshahi division, Bangladesh

**DOI:** 10.1186/s12905-022-01665-6

**Published:** 2022-03-23

**Authors:** Md. Abu Tal Ha, Md. Zakiul Alam

**Affiliations:** Department of Population Sciences, University of Dhaka, Dhaka, 1000 Bangladesh

**Keywords:** Menstrual hygiene management practice, Menstrual hygiene, Adolescent girls, Bangladesh

## Abstract

**Introduction:**

Adolescence is a critical period characterized by significant physical, emotional, cognitive, and social changes, including the monthly occurrence of menstruation of adolescent girls. Despite being an inevitable natural event, most societies consider menstruation and menstrual blood as taboos and impure. Such consideration prevents many adolescent girls from proper health education and information related to menstrual health, which forces them to develop their ways of managing the event. This study attempted to explore the pattern, the urban–rural differences, and the determinants of menstrual hygiene management practices (MHMP) among adolescent girls in the Rajshahi division, Bangladesh.

**Methodology:**

Using a cross-sectional study design with multistage random sampling, we collected data from 586 adolescent girls (aged 14–19 years) from the Rajshahi division of Bangladesh. The MHMP was measured using eight binary items, where the value from zero to five as ‘bad,’ six as ‘fair,’ and seven-eight as ‘good’ practices. Finally, we employed bivariate analysis and multinomial logistic regression analysis.

**Findings:**

Only 37.7% continuously used sanitary pads. Among the cloth users, nearly three-fourths reused cloths, and about 57% used water and soap to wash them. About 49% changed menstrual absorbent, and 44% washed their genitalia three times daily. About 41% used water only to wash genitalia, and 55% buried sanitary materials under the soil. Around 36.9% of the girls practiced bad, 33.4% fair, and 29.7% good menstrual management. We found significant differences in MHMP among adolescent girls between urban and rural areas (32.3% vs. 27.7% good users, *p* ≤ *0.05*). Multinomial logistic regression found that place of residence, age, family size, parental education, and age at first menstruation were the significant determinants of MHMP.

**Conclusion:**

Although there are some cases of sanitary pad use, still menstrual hygiene management is unhealthy in most cases. The continuous supply of sanitary pads at affordable cost, change in existing social norms about menstruation, proper education, information, and services are essential for achieving health-related SDG goals in both rural and urban areas of Bangladesh.

## Introduction

Adolescence is a critical period in women’s lives characterized by first menstruation, a natural and beneficial biological event, and significant physical, emotional, cognitive, and social changes [1, 2]. Despite being an inevitable and natural process, most societies consider menstruation a taboo [1–6]. Many of the norms and stigma associated with the event are based on discriminatory gender roles and cultural restrictions, making it a silent and invisible issue [1–6]. As a result, it prevents many adolescent girls from receiving proper menstrual health and hygiene-related information and education. Furthermore, it also exposes them to challenges of managing menstruation and menstrual blood properly and forces them to develop their ways of managing it depending on existing traditional and cultural beliefs, level of knowledge on menstruation, and personal preferences [1–6].

In a country like Bangladesh, mothers and other female relatives are the primary sources of information on menstruation; however, they can provide very little information, which is often misconceptions, thereby affecting adolescent girls’ response to menstrual management [7–10]. Studies show that unhealthy practices of menstrual management among adolescent girls are highly prevalent in Bangladesh [1, 7, 11, 12]. Globally, poor menstrual management affects girls’ school attendance and academic progress through psychological (for example, discomfort, high stress, fear of leakage of menstrual blood, and fear of leaving signs of menstrual blood inside the school latrine) and physical (for example, Dysmenorrhoea, Headache, and excessive bleeding) factors [2, 12, 13]. At the same time, it affects their maternal and reproductive health through increased risk of reproductive tract infections (RTI), sexually transmitted diseases (STD), Human Papillomavirus (HPV) infection, and adverse pregnancy outcomes [1, 2, 14]. Understanding the menstrual hygiene management practices (MHMP) of adolescent girls is therefore crucial for informing strategies to promote equitable education, gender equality, women’s empowerment, health, and environment in line with the Sustainable Development Goals (SDGs) [10].

Despite being a significant public health issue, there is limited understanding of the extent of proper MHMP in low and middle-income countries, including Bangladesh [11]. Studies on menstrual management practices conducted in Bangladesh have, for instance, focused on narrow topics such as the prevalence of the practices or the relationship between menstruation and girls’ school attendance, with no consideration of the broader dimensions of menstrual hygiene management and its socioeconomic determinants [1, 7, 8, 10, 12]. This paper examines the menstrual management practices of adolescent girls in the Rajshahi Division (randomly selected) of Bangladesh and the socioeconomic determinants of such practices.

## Data and methods

### Data collection process

**Fig. 1:**
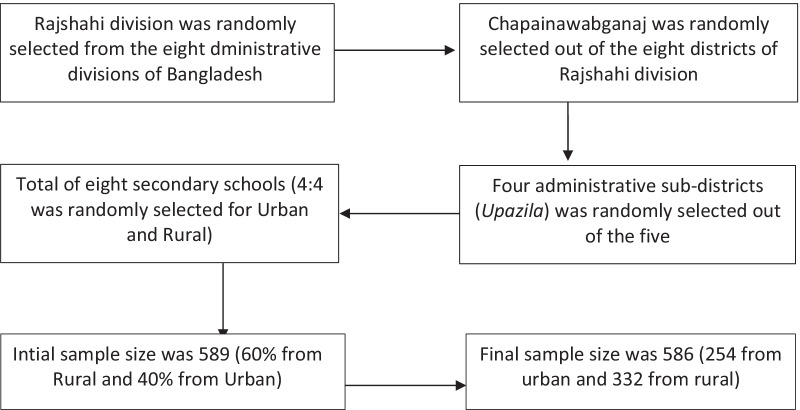
Process of sample selection for the study

We used a multistage random sampling procedure to identify the research location. In the first stage, we randomly selected the study area, the Rajshahi division, among Bangladesh's eight administrative divisions. One of the administrative districts, Chapainawabganaj, was then randomly selected among the eight districts of the Rajshahi division. In the third stage, four administrative sub-districts (*Upazila* in Bangladesh) were selected randomly out of the five sub-districts. In the final stage, eight secondary schools (four located in the urban area and the other four in the rural area) were randomly selected with the help of key informants to locate the schools., The target sample size was 589 adolescent girls in order to detect 10% prevalence of use of sanitary pad (based on the 2014 Bangladesh National Hygiene Baseline Survey) at a 95% confidence interval, 5% non-response rate, and 1.5 design effect for homogeneity among learners sampled from the same school. The current cross-sectional study was conducted on 17–25 November 2018 among adolescent girls aged 14 to 19 years studying in eight secondary schools in the Rajshahi Division of Bangladesh. In each school, an average of 37 adolescent girls (total sample divided by two classes of each school) in Standards 9 and 10 were randomly selected from the registrar. The initial target was to have 60% of the sample from rural areas and 40% from urban areas, as most (more than 60%) of the population lives in rural areas. However, the final sample size stands at 586 adolescent girls comprising 57% from rural areas and 43% from urban areas (Fig. 1).

Adolescent girls studying in Standard 9 and 10 were targeted; they were considered mature enough to talk about a socially sensitive issue like menstruation and could provide their consent for participation in the research. Before the commencement of data collection, permission was obtained from the head of each school over the phone after informing the study's objectives. As permission was received, we collected detailed information of the potential respondents from those schools, randomly selected them, contacted their parents, and managed to receive their permission as the potential participants were minors. After getting permission from both heads of institutions and parents, one of the female teachers from each school was trained on the data collection process to oversee. On the interview day, randomly selected girls from Standard 9 and 10 were gathered in a classroom and were seated in-class environment. A questionnaire was given to students who voluntarily agreed to participate in the study after a short description of objectives and roles as respondents. After that, participants self-administered the questionnaire. The responsible female teacher collected the completed questionnaires.

### Variable definitions and measurements

#### Outcome variable

The dependent variable, “Menstrual Hygiene Management Practice (MHMP),” had three categories, namely ‘good,’ ‘fair,’ and ‘bad.’ A total of 8indicators, identified based on existing literature [7, 12, 15–20], were used to measure menstrual hygiene management practices (Table 1).

**Table 1:** Measures of menstrual hygiene management practices

Serial number	Indicators	Positive responses = 1	Negative responses = 0
1	Absorbent used for managing menstrual blood	Sanitary pad, new cloth, Use of sanitary pad and new cloth interchangeably**	Old cloth
1.1	Whether the respondent washed the cloth before re-using	Yes	No
1.2	Material/s used for washing clothes before re-using	Water and soap, Water and antiseptic, Water, soap and antiseptic**	Water only
1.3	Drying place of washed cloth	Open sunny places	Keeping in bed/other clothes, closed and shaded places, open but shaded places
1.4	Storing place of dried cloth before re-using	With other clothes	Within the bathroom, hidden places within the bedroom
2	Frequency of changing absorbent in a day	Three or more times a day	Less than three times a day
3	Whether the respondent washed genitalia during menstruation	Yes	No
4	frequency of washing genitalia in a day during menstruation	Four or more times a day	Less than four times a day
5	Materials used for washing genitalia during menstruation	Water and soap, Water soap and antiseptic**	Water only
6	Whether the respondent took a bath during menstruation	Yes	No
7	Frequency of taking a bath during menstruation	At least a single time a day	Irregular bathing or less than one time a day
8	Disposal of sanitary materials after using	Burial under the soil, placing in a dustbin with another wastage, placing in toilet’s commode and then flushing water on it**	Throwing into the river, throwing into the pond, throwing in open places

*Indicators 1.1 to 1.4 for cloth users; the respondent who scored positive in each response were coded as positive for old cloth users and counted positive response for indicator 1

**Practicing any one of these to manage menses was considered a positive response

For measurement purposes, healthy practices for each indicator were coded one, and unhealthy practices as zero. The first indicator was related to absorbent use for managing menstrual blood, where the use of sanitary pads and new cloth was regarded as healthy practices. However, the questions on washing cloth before re-using, materials used to a washcloth, place of drying and storing washed cloth (indicators 2.1 to 2.4 in Table 1) were only asked those who used the cloth to manage their menses [12, 19–21]. The positive response of all the four indicators about using old cloth was also considered healthy practices for indicator one. The other seven indicators included frequency of changing absorbent, washing of genitalia during menstruation, frequency of washing genitalia, the material used for washing genitalia, taking a bath during menstruation, frequency of bathing during menstruation, and ways of disposal of sanitary materials [7, 12, 15–20] and were applicable for all participants. Therefore, a score for the sum of eight indicators was used to measure MHM practices, with values ranging from zero to eight. The indicator was then categorized into bad (score of zero to five: up to 50%), fair (score of six: 50–75%), and good (score of seven and eight: 75–100%) MHM practices.

#### Predictors

Socioeconomic and demographic variables were used as the independent variables, including age, family size, religion, wealth index, fathers’ and mothers’ education, fathers’ and mothers’ income, age of first menstruation, social connectivity, and information on menstruation before reaching menarche based on the existing literature [1, 10, 12, 15, 19, 21–29]. Age was categorized as ≤ 15 and ≥ 16 years old considering the year of schooling (class 9 and 10) and mean age (15.1) of the participants. Since mother and other female relatives were the primary sources of information on menstruation in Bangladesh [8], adolescent girls from small families may have a lower chance of receiving menstrual information; as the number of female relatives tends to be lower in these families. Thus, we decided to assess the relation between family size and menstrual hygiene management. Families with five or fewer members were defined as small, and more than five members as large families based on the average family size (4.4) in Bangladesh [30].

We used ten items on household possessions to generate an indicator of an individual’s family wealth status. Questions included flooring type of the respondent’s household, electricity connection, steel/wooden Almirah, smartphone, toilet, type of toilet, color television, refrigerator, air conditioner, and availability of transport used for a non-business purpose. All variables had binary responses (yes or no) except the type of toilet and transport. Positive responses were assigned value one, and negative answers were attributed zero. Type of toilet facility and transport were recoded into dummy variables. The availability of a motorcycle and/or cars/micro/bicycle was considered a positive response. Similarly, the availability of open and/or *kancha* (mud) toilets was considered a negative response, while sanitary toilets (with or without water slab) were considered a positive response. One was attributed for both variables for a positive response and zero for a negative response. The principal component analysis (KMO = 0.71) was used to measure the wealth index and categorized poor (bottom 40%), middle (next 40%), and rich (top 20%) following existing literature [24].

The perceived socioeconomic class was measured by two questions that asked participants about their perceptions of the specific class to which they belonged, with the response of very poor, poor, middle, upper, and uppermost. For analytical purposes, those who responded very poor and poor were assigned value one, the middle was attributed two, and the upper and uppermost were attributed three. The two variables were then summed to generate a variable with values ranging from two to six. Respondents attaining value 1–2 were defined as low, 3–4 as middle, and 5–6 were defined as a high socioeconomic class. As existing literature suggests, although there is a high unmet need for sanitary pads, many adolescent girls and women do not use them because of high costs [8]. At the same time, many women and adolescent girls still consider sanitary pads as a luxury item [31]. Thus, we incorporated this variable to assume that perceived socioeconomic class may affect sanitary pad use and other menstrual hygiene management practices. We also measured the fathers' income as low (up to BDT 10,000), middle (BDT 10,001 to 20,000), and high (BDT 20,001+); mothers’ income as yes and no.

We used three variables to measure respondents' social connectivity: physical mobility, passing the time with friends outside the school, and internet use. Respondents who could travel more than one kilometer alone from home during the daytime without parental consent were defined as physically mobile, and those who could travel less than one kilometer as non-mobile. We considered a minimum distance of one kilometer based on the assumption that sanitary pads may not be available within this one square kilometer area, thus limiting the chance of buying sanitary pads by respondents themselves. Physical mobility was attributed value one, and non-mobility was attributed zero. Similarly, passing the time with friends other than school period and using the internet regularly had two possible responses; yes and no; positive answers were assigned value one, and negative answers were assigned zero. The three variables were summed to generate a score with values ranging from zero to three. Those who scored zero were considered not socially connected, those with 1–2 as moderately connected, and those scoring three as highly connected.

Respondents’ perception towards pad use was measured using a set of four statements: (1) *use of old clothes rather than pads to manage menses may increase the risk of reproductive tract infections*, (2) *use of sanitary pads to manage menses may prevent reproductive tract infections*, (3) *use of sanitary pads helps to regular menstruation*, and (4) *use of sanitary pads protects one from the fear of unwanted drop out of it*. Each statement had five options; strongly agree, agree, do not know/not sure, disagree, and strongly disagree. Responses ‘agree’ and ‘strongly disagree’ were coded as positive responses, while the rest were negative responses.

### Analytical approach

We used cross-tabulation with the Chi-square test to examine urban–rural differences in various menstrual hygiene practices. We then estimated a multinomial logistic regression model to examine the factors associated with hygiene management practices. The results from cross-tabulations are presented as percentages, while those from multinomial logistic regression analysis are presented as [coefficient estimates/relative risk ratios (RRR)] with 95% confidence intervals (CI).

## Findings

### Socio-demographic characteristics of respondents

Table 2 represents the socio-demographic characteristics of respondents. Data show that the mean age of the respondents in this study was 15.5 (standard deviation is 0.71 years). Out of 586 girls, 254 (43.3%) lived in urban areas and the rest in rural areas. Most (71.8%) girls lived in small families with five members or fewer. Data regarding parents’ education indicated that 7.8% of fathers and 5.1% of mothers did not have formal education. The majority of the respondents (81.1%) were from middle-class families. About a third (32.8%) of the adolescent girls did not have social connections, while 31.7% reported having high social connectivities.

**Table 2:** Background characteristics of respondents

Background characteristics	Urban, n (%)	Rural, n (%)	Total, n (%)	*P*-value
**Age**				0.005
≥ 15	117 (46.1)	192 (57.8)	309 (52.7)	
≤ 16	137 (53.9)	140 (42.2)	277 (47.3)	
**Family size**				0.944
Small	181 (71.3)	240 (72.3)	421 (71.8)	
Large	73 (28.7)	92 (27.7)	165 (28.2)	
**Religion**				< 0.001
Islam	237 (93.3)	306 (92.2)	543 (92.7)	
Hindu	17 (6.7)	26 (7.8)	43 (7.3)	
**Wealth Index**				< 0.001
Poor	77 (30.3)	159 (49.9)	236 (40.3)	
Middle	123 (48.4)	110 (33.1)	233 (39.8)	
Rich	54 (21.3)	63 (19.0)	117 (20.0)	
**Father’s education**				< 0.001
Illiterate	6 (2.4)	40 (12.0)	46 (7.8)	
Primary	111 (43.7)	103 (31.0)	214 (36.5)	
Secondary	69 (27.2)	121 (36.4)	190 (32.4)	
Higher Secondary and above	68 (26.8)	68 (20.5)	136 (23.2)	
**Mother’s education**				0.009
Illiterate	7 (2.8)	23 (6.9)	30 (5.1)	
Primary	76 (29.9)	89 (26.8)	165 (28.2)	
Secondary	147 (57.9)	190 (57.2)	337 (57.5)	
Higher Secondary and above	24 (9.4)	30 (9.0)	54(9.2)	
**Father’s income**				< .001
Low	97 (38.2)	194 (58.4)	291 (49.73)	
Middle	116 (45.7)	115 (34.6)	231 (39.4)	
High	41 (16.1)	23 (6.9)	64 (10.9)	
**Mother’s income**				0.911
No	190 (74.8)	247 (74.4)	437 (74.6)	
Yes	6 (25.2)	85 (25.6)	149 (25.4)	
**Perceived socioeconomic class**				< 0.001
Lower	27 (10.6)	24 (7.2)	51 (8.7)	
Middle	216 (85.0)	259 (78.0)	475 (81.1)	
Upper	11 (4.3)	49 (14.8)	60 (10.2)	
**Social connectivity**				0.218
No connection	74 (29.1)	118 (35.5)	192 (32.8)	
Moderate connection	98 (38.6)	110 (33.1)	208 (35.5)	
High connection	82 (32.3)	104 (31.3)	186 (31.7)	

### Menstrual knowledge of respondents

Results on menstrual knowledge are presented in Table 3. The mean age at first menstruation was 12.8 (standard deviation of 0.97 years), slightly higher in rural than urban areas (12.9 and 12.6 years, respectively; *p* = 0.008). Three-quarters (75%) of the respondents reported that they received information about menstruation before reaching menarche, while the rest did not receive any information before their first menstruation. Sources of information about menstruation were mothers (42.1%), female friends (22.9%), female teachers (18.5%), sisters (13.2%), and others (3.2%). Results further show that half (50.2%) of the girls had positive perceptions about the use of sanitary pads. There were statistically significant variations in availability and sources of information and perception about the use of sanitary pads between adolescent girls in urban and rural settings. For instance, urban adolescents were more likely to receive information before menarche, specifically from female teachers, than their counterparts.

**Table 3:** Distribution of adolescent girls by knowledge, perceptions, and experiences of menstruation

Background characteristics	Urban, n (%)	Rural, n (%)	Total, n (%)	*P*-value
**Age of first menstruation**				0.008
≥ 12	104 (40.9)	98 (29.5)	202 (34.5)	
≤ 13	150 (59.1)	234 (70.5)	384 (65.5)	
**Information availability before reaching menarche**				0.024
Yes	203 80.2)	236 (71.1)	439 (75.0)	
No	50 (19.8)	96 (28.9)	146 (25.0)	
**Source of information**				< 0.001
Mother	77 (38.3)	105 (45.5)	182 (42.1)	
Female friend	38 (18.9)	61 (26.4)	99 (22.9)	
Sister	21 (10.4)	36 (15.6)	57 (13.2)	
Teacher	59 (29.4)	21 (9.1)	80 (18.5)	
Others	6 (3.0)	8 (3.5)	14 (3.2)	
**Perception of sanitary pad use**				< 0.001
Positive	103 (40.6)	191 (57.5)	294 (50.2)	
Negative	151 (59.4)	141 (42.5)	292 (49.8)	

### Menstrual hygiene management practices of adolescent girls

Table 4 presents the findings and shows that the highest 37.7% of adolescent girls used sanitary pads and 27.1% used old/new cloth only to manage their menstrual blood. The use of absorbent does not vary statistically (*p* = 0.47) between urban and rural areas. Among the cloth users (n = 363, including occasionally cloth users), nearly three-fourths (71.1%) reused the same cloth, and almost all (97.7%) washed these cloths before reusing. Water and soap were used as the main ingredient (57%) in urban and rural areas. In 51.6% of cases, the washed cloth was dried in open sunny places, and after drying, nearly three-fourths (74.7%) of them were stored in hidden places in the room. Nearly half (48.5%) of girls changed menstrual materials three times a day, and only 0.4% changed it four or more times. These practices did not vary based on place of residence. Almost all (98.8%) washed their genitalia during menstruation. Data show that 44.2% of girls washed their genitalia three times, 33.7% four or more times, and 8.4% once a day. Around 41% of girls used only water to wash genitalia, which was higher in urban areas than rural areas. Both washing frequency and materials varied significantly between the two areas. Urban girls were more likely to wash their genitalia four or more times a day than rural girls, while rural girls were less likely to use water only to wash external genitalia than urban girls. More than three-fourths of the girls (77.5%) took a bath regularly during menstruation. After use, 55.3% of the girls buried their sanitary materials under the soil, and 20.2% threw them in the pond or river—the practice of disposing of sanitary materials varied between the two areas. Rural girls were more likely to throw their used sanitary materials into ponds or rivers; on the other hand, more urban girls buried them under the soil.

**Table 4:** Menstrual hygiene management practice of adolescent girls

Indicators of MHM practice	Urban, n (%)	Rural, n (%)	Total, n (%)	*P*-value
**Absorbent used**				0.470
Sanitary pad	106 (41.7)	114 (34.7)	220 (37.7)	
Sometimes pad sometimes cloth	87 (34.3)	118 (35.9)	205 (35.2)	
Old cloth	40 (15.7)	69 (21.0)	109 (18.7)	
New cloth	21 (8.3)	28 (f8.5)	49 (8.4)	
**Reuse of cloth**				0.033
No	51 (34.9)	53 (24.8)	104 (28.9)	
Yes	95 (65.1)	161 (72.2)	256 (71.1)	
**Washing of cloth before reuse**				a
Yes	92 (93.9)	163 (100.0)	255 (97.7)	
No	6 (6.1)	00 (0.0)	6 (2.3)	
**Washing materials**				0.057
Water and soap	46 (47.9)	100 (62.1)	146 (57.0)	
Water and antiseptic	12 (12.5)	21 (13.0)	33 (12.9)	
Water soap and antiseptic	31 (32.3)	27 (16.8)	58 (22.7)	
Water only	7 (7.3)	12 (7.5)	19 (7.4)	
**Place of drying washed cloth before reuse**				a
By keeping in bed or other clothes	3 (3.1)	9 (5.7)	12 (4.7)	
In shaded and closed place	23 (23.7)	45 (28.3)	68 (26.6)	
In shaded but open place	13 (13.4)	31 (19.5)	44 (17.2)	
In open sunny place	58 (59.8)	74 (46.5)	132 (51.6)	
**Place of storing dried cloth**				0.157
Hidden place within the room	78 (81.3)	111 (70.7)	189 (74.7)	
With other clothes	9 (9.4)	26 (16.6)	35 (13.8)	
Within the bathroom	9 (9.4)	20 (12.7)	29 (11.5)	
**Frequency of changing absorbent**				a
Less than one time a day	00 (0.0)	12 (3.7)	12 (2.1)	
One time per day	23 (9.3)	33 (10.2)	56 (9.8)	
Two times per day	96 (38.9)	128 (39.5)	224 (39.2)	
Three times per day	126 (51.0)	151 (46.6)	277 (48.5)	
Four or more times per day	2 (0.8)	00 (0.0)	2 (.4)	
**Genitalia washing during menstruation**				a
Yes	246 (99.6)	314 (98.1)	560 (98.8)	
No	1 (0.4)	6 (1.9)	7 (1.2)	
**Genitalia washing frequency**				< 0.001
One time per day	18 (7.3)	29 (9.2)	47 (8.4)	
Two times per day	20 (8.1)	57 (18.2)	77 (13.7)	
Three times per day	107 (43.3)	141 (44.9)	248 (44.2)	
Four or more times per day	102 (41.1)	87 (27.7)	189 (33.7)	
**Genitalia washing material**				a
Water only	117 (47.4)	112 (35.3)	229 (40.6)	
Water and soap	67 (27.1)	151 (47.6)	218 (38.7)	
Water soap and antiseptic	62 (25.1)	54 (17.0)	116 (20.6)	
Others	1 (0.4)	00 (0.0)	1 (0.2)	
**Taking a bath during menstruation**				a
Yes	250 (100.0)	324 (99.4)	574 (99.7)	
No	00 (0.0)	2 (0.6)	2 (0.3)	
**Frequency of bathing during menstruation**				a
Less than one time a day	60 (24.6)	56 (17.2)	116 (20.4)	
One time per day	181 (74.2)	260 (80.0)	441 (77.5)	
Two or more time per day	3 (1.2)	9 (2.8)	12 (2.1)	
**Ways of disposing of sanitary materials**				a
Throwing into pond or river	10 (4.0)	106 (32.6)	116 (20.2)	
Throwing in open places	1 (0.4)	6(1.8)	7 (1.2)	
Burying under the soil	192 (77.4)	125 (38.5)	317 (55.3)	
Throwing into the dustbin with other wastage	37 (59.7)	25 (40.3)	62 (10.8)	
Throwing into the commode and pouring water	4 (1.6)	60 (18.5)	64 (11.2)	
Others	4 (1.6)	03 (0.9)	7 (1.2)	

‘a’ refers that the assumption of the chi-square test did not fulfill for this variable

### Barriers to continuous use of sanitary pad

Table 5 presents barriers to the continuous use of sanitary pads. Among the sanitary pad users, including mixed users (sometimes cloth and sometimes pad users), respondents (45.5%) and their parents (25.1% by mothers and 20.6% by fathers) were the primary buyers of sanitary pads. However, these sources varied based on place of residence. More girls in urban areas relied on their parents to buy sanitary materials, while more rural girls themselves bought them. More than one-third of girls felt shy to buy sanitary materials from male shopkeepers (36.1%), and this shyness increased in the presence of a male in the shop other than the shopkeeper (60.1%). More than three-fourths (76.1%) of adolescent girls could not use sanitary pads continuously due to their absence at home during their menstruation. In this circumstance, they either used old/new clothes (66.2%) or borrowed sanitary pads (28.4%) from others or took other measures to manage this. Among all-time cloth users, including old and new cloth, 71.8% wished to use sanitary pads but could not because of high cost (35.6%), feeling of embarrassment to buy sanitary pads (37.5%), unavailability of sanitary pads at nearby shops (10.6%) and other (16.3%) causes. Adolescent girls' unwilling to use sanitary pads mentioned relaxation to use cloths (60%), unnecessary money expending (27.5%), rashes (10%), and other (2.5%) causes as a reason.

**Table 5:** Barriers to continuous sanitary pad use

Selected barriers to continuous sanitary pad use	Urban, n (%)	Rural, n (%)	Total n, (%)	*P*-value
**Sanitary pad buyer**				< 0.001
Respondent herself	81 (40.5)	115 (49.8)	196 (45.5)	
Mother	53 (26.5)	55 (23.8)	108 (25.1)	
Father	58 (29.0)	31 (13.4)	89 (20.6)	
Sister	6 (3.0)	20 (8.7)	26 (6.0)	
Brother and or friends and or others	2 (1.0)	10 (4.3)	12 (2.8)	
**Feeling of shyness to buy sanitary materials from a male shopkeeper**				0.010
Yes	21 (24.7)	53 (44.2)	74 (36.1)	
No	64 (75.3)	67 55.8)	131 (63.9)	
**Feeling of shyness to buy a sanitary pad in the presence of male/s other than the shopkeeper**				< 0.001
Yes	38 (45.8)	81 (70.4)	119 (60.1)	
No	45 (54.2)	34 (29.6)	79 (39.9)	
**Total reserve of the sanitary pad at home during menstruation**				0.141
No	155 (78.3)	164 (74.2)	319 (76.1)	
Yes	43 (21.7)	57 (25.8)	100 (23.9)	
**Measures taken in the absence of sanitary pad during menstruation**				0.047
Uses old cloth	32 (20.6)	59 (36.4)	91 (28.7)	
Uses new cloth	67 (43.2)	52 (32.1)	119 (37.5)	
Borrows sanitary pad from other	46 (29.7)	44 (27.2)	90 (28.4)	
Others	10 (6.5)	7 (4.3)	17 (5.4)	
**Non-pad users desire for using sanitary pad during menstruation**				0.203
Yes	37 (78.7)	70 (68.6)	107 (71.8)	
No	10 (21.3)	32 (31.4)	42 (28.2)	
**Cause of not using sanitary pad currently despite being desired**				0.271
Pads are very costly	15 (44.1)	22 (31.4)	37 (35.6)	
Feels embarrassed to buy sanitary pads	13 (38.2)	26 (37.1)	39 (37.5)	
Shop where pads are available is at long distance	3 (8.8)	8 (11.4)	11 (10.6)	
Others	3 (8.8)	14 (20.0)	17 (16.3)	
**Cause of not willing to use sanitary pads**				0.498
Feels relaxed to use cloth	07 (53.8)	17 (63.0)	24 (60.0)	
Sanitary pads cause unnecessary money spending	04 (30.8)	07 (25.9)	11 (27.5)	
Sanitary pad causes rashes or other problems	01 (7.7)	03 (11.1)	4 (10.0)	
Others	01 (7.7)	00 (0.0)	1 (2.5)	

### Association between socioeconomic factors and menstrual hygiene management practices

The classification of menstrual hygiene management practices indicated that 36.9% of respondents followed bad, 33.4%, and 29.7% followed fair and good practices (Table 6). Variations in menstrual hygiene management practices show that urban adolescent girls had more good and fair practices (*p* = 0.05) than rural girls. Similarly, girls from small families had more good practices than girls from large families (*p* = 0.030). Parental education, parental income, and age at first menstruation were also statistically significant (Table 6). At the same time, religion, wealth index, perceived socioeconomic class, information before menstruation, perception toward pad use, and social connectivity were not significantly associated with menstrual hygiene management practices. Girls who experienced first menstruation at or after thirteen years of age were more likely to practice good and fair management than those who experienced first menstruation at earlier ages.

**Table 6:** Bivariate associations between socioeconomic-demographic factors and menstrual hygiene management practices

Variable	Bad, n = 216 (%)	Fair, n = 196 (%)	Good, n = 174 (%)	*P*-value
**Place of residence**				0.050
Urban	83 (32.7)	89 (35.0)	82 (32.3)	
Rural	133 (40.1)	107 (32.2)	92 (27.7)	
**Age**				0.002
≥ 15	98 (31.6)	102 (32.9)	110 (35.5)	
≤ 16	118 (42.8)	94 (34.1)	64 (23.2)	
**Religion**				0.654
Islam	200 (36.8)	184 (33.9)	159 (29.3)	
Hindu	16 (27.2)	12 (27.9)	15 (34.9)	
**Family size**				0.030
Small	144 (34.3)	139 (33.1)	137 (32.6)	
Large	72 (43.4)	57 (34.3)	37 (22.3)	
**Wealth quintile**				0.149
Poor	81 (34.3)	86 (36.4)	69 (29.2)	
Middle	94 (40.3)	64 (27.5)	75 (32.2)	
Rich	41 (35.0)	46 (39.3)	30 (25.6)	
**Father’s education**				< 0.001
Illiterate	18 (39.1)	18 (39.1)	10 (21.7)	
Primary	89 (41.6)	74 (34.6)	51 (23.8)	
Secondary	77 (41.0)	60 (31.9)	51 (27.1)	
HSC and/or Above	32 (23.2)	44 (31.9)	62 (44.9)	
**Mother’s education**				0.007
Illiterate	5 (16.7)	13 (43.3)	12 (40.0)	
Primary	69 (41.8)	63 (38.2)	33 (20.0)	
Secondary	127 (37.9)	101 (30.1)	107 (31.9)	
HSC and/or Above	15 (26.8)	19 (33.9)	22 (39.3)	
**Father’s income**				0.001
Low	120 (41.2)	102 (35.1)	69 (23.7)	
Middle	76 (32.9)	65 (28.1)	90 (39.0)	
High	20 (31.3)	29 (45.3)	15 (23.4)	
**Mother’s income**				0.745
No	161 (36.8)	143 (32.7)	133 (30.4)	
Yes	55 (39.6)	53 (25.6)	41 (27.5)	
**Perceived socioeconomic class**				0.099
Low	11 (21.6)	22 (43.1)	18 (35.3)	
Middle	180 (38.0)	151 (31.9)	143 (30.2)	
High	25 (41.0)	23 (37.7)	13 (21.3)	
**Social connectivity**				0.116
No	79 (41.1)	67 (34.9)	46 (24.0)	
Moderate	80 (38.5)	65 (31.3)	63 (30.3)	
High	57 (30.6)	64 (34.4)	65 (34.9)	
**Age of first menstruation**				0.010
≥ 12	91 (45.0)	61 (30.2)	50 (24.8)	
≤ 13	125 (32.6)	135 (35.2)	124 (32.3)	
**Information availability before menstruation**				0.866
No	56 (38.4)	49 (33.6)	41 (28.1)	
Yes	160 (36.4)	147 (33.4)	133 (30.2)	
**Perceptions towards Pad use**				0.450
Negative	101 (34.4)	102 (34.7)	91 (31.0)	
Positive	115 (39.4)	94 (32.2)	83 (28.4)	
**Total**	216 (36.9)	196 (33.4)	174 (29.7)	

Only significant variables (p-value of 0.05 or less) from the bivariate analysis were entered into regression analysis (Table 7). The results show that the girls aged up to 15 years were less likely to have bad menstrual hygiene management practices (RRR: 0.50; 95% CI: 0.32–0.80, *P* = 0.004) than if they did not have good management practices than the girls aged 16 and above years. Urban girls were less likely (RRR: 0.61; 95% CI: 0.38–0.98, *P* = 0.045) to have bad menstrual hygiene management practices if they did not have good management practices compared to those from rural areas. Adolescent girls from small families were significantly less likely to have bad (RRR: 0.52; 95% CI: 0.31–0.86) menstrual hygiene management practices if they did not have good management practices compared to those from large families. Girls whose fathers had no formal (RRR: 2.28; 95% CI: 1.16–9.28, *P* = 0.025), primary (RRR: 3.56; 95% CI: 1.84–6.88, *P* < 0.001) and secondary education (RRR: 3.11; 95% CI: 1.67–5.82, *P* < 0.001) were more likely to have bad menstrual hygiene management practices compared to those whose fathers had HSC or higher levels of education. Girls who experienced their first menstruation at 12 years or earlier were 66% more likely to practice bad menstrual hygiene management than those who experienced their first menstruation at 13 years or older (RRR: 1.66; 95% CI: 1.05–2.63, *P* = 0.030).

**Table 7:** Relative risk ratios from multinomial logistic regression analysis examining factors associated with menstrual hygiene management practices among adolescent girls

Variable Name (Ref = Good)	Bad		Fair	
	RRR (95% CI)	Sig	RRR (95% CI)	Sig
**Place of residence (Ref = Rural)**				
Urban	0.61 (0.38, 0.98)	0.045	0.83 (0.52, 1.33)	0.436
**Age (Ref = ≥ 16)**				
≥ 15	0.50 (0.32, 0.80)	0.004	0.71 (0.44, 1.13)	0.146
**Family size (Ref = Large)**				
Small	0.52 (0.31, 0.86)	0.011	0.66 (0.40, 1.10)	0.115
**Religion (Ref = Hindu)**				
Islam	1.31 (0.59, 2.90)	0.510	1.51 (0.65, 3.49)	0.338
**Fathers’ education (Ref = HSC and/or Above)**				
Illiterate	3.28 (1.16, 9.28)	0.025	1.84 (0.67, 5.03)	0.238
Primary	3.56 (1.84, 6.88)	< 0.001	1.96 (1.04, 3.71)	0.039
Secondary	3.11 (1.67, 5.82)	< 0.001	1.64 (0.90, 3.01)	0.108
**Mothers’ education (Ref = HSC and/or Above)**				
Illiterate	0.20 (0.05, 0.83)	0.027	0.81 (0.23, 2.79)	0.738
Primary	1.07 (0.41, 2.75)	0.895	1.45 (0.59, 3.61)	0.420
Secondary	0.97 (0.44, 2.16)	0.943	0.93 (0.44, 1.99)	0.860
**Fathers’ income (Ref = High)**				
Low	0.91 (0.39, 2.13)	0.827	0.62 (0.28, 1.39)	0.244
Middle	0.50 (0.23, 1.12)	0.094	0.32 (0.15, 0.69)	0.003
**Perceived socioeconomic class (Ref = High)**				
Low	0.32 (0.10, 0.98)	0.047	0.50 (0.18, 1.41)	0.189
Middle	0.69 (0.32, 1.52)	0.358	0.54 (0.25, 1.18)	0.124
**Age of first menstruation (Ref = ≤ 13)**				
≥ 12	1.66 (1.05, 2.63)	0.030	1.07 (0.67, 1.72)	0.771
**Model summary**				
AIC of the null model	1025.2			
AIC of final model	992.1			
Cox and Snell R square	0.153			
Nagelkerke R square	0.172			

*RRR* relative risk ratios, *CI* confidence interval, *Sig.* significance (probability value)

## Discussions

The main objectives of this study were to explore the menstrual hygiene management practices, the urban–rural differences in the practices, and the determinants of such practices. Our findings indicate that the mean age of respondents and the mean age of first menstruation in the study were 15.5 and 12.8 years, respectively. The mean age at first menstruation was slightly higher in rural (12.9) areas than urban areas (12.6). These findings are consistent with similar studies [7, 10, 19, 32–34]. Proper education and information on menstruation before reaching menarche are the right to information of adolescent girls and are crucial for healthy menstrual management. A cross-sectional study conducted by Alam et al. (2017) shows that 64% of girls did not know about menstruation before reaching menarche [10]. The Bangladesh National Hygiene Baseline Survey 2014 also described that only 36% were informed about menstruation among the students before reaching menarche [7]. Compared to these studies, our study indicates that about 75% of the respondents received information about menstruation before reaching menarche, which indicates an increase in getting menstrual information before reaching menarche.

Before reaching menarche, getting information regarding menstruation was positively but not significantly associated with menstrual hygiene management practices. Like other studies, this study also found that mothers and sisters together were the two primary sources of menstruation-related information [7, 10, 19, 35, 36]. This finding is important because mothers with a lower level of knowledge can transfer very little regarding menstruation and often transfer misconceptions to their girl child [9]. On the other hand, high literacy and slight inhibition of mothers sharing their accurate knowledge to their daughters can positively affect adolescent girls’ conception and healthy management of menstruation over generations [19].

The absorbent used for managing menstrual blood is a primary health concern. The reuse of cloths without maintaining proper hygiene may increase the risk of reproductive tract infections [19]. The prevalence of using sanitary pads was 37.7%, significantly higher than previous studies conducted in Bangladesh [7, 8, 10, 12, 31]. According to our study, among the cloth users, including sometimes sanitary pads and sometimes cloth users, 71.1% reused cloths for absorbing menstrual blood, and 97.7% washed those materials before reusing; among them, 57% used water and soap, and 7.4% used water only. These findings were consistent with similar studies [1, 7, 10, 15, 19, 25, 37]. Re-using of clothes may even be better when proper sanitization is maintained as cotton cloths are reusable, readily available, and more environment-friendly than commercial sanitary pads [38]. However, drying sanitary materials in open and sunny places before re-using (51.6%) was significantly higher. Moreover, 74.7% of the respondent stored their dried clothes in hidden places within the room, and 13.8% with other cloths for reuse, and these findings differed from other studies [7, 12, 15].

Regular changing sanitary materials is a prerequisite for maintaining good menstrual hygiene management. Although there is no hard and fast rule of changing sanitary materials during menstruation, it depends mostly on blood flow, varying from person to person. However, it is suggested to change absorbent at least every four to eight hours during the menstrual cycle [7]. We considered changing sanitary materials at least three times a day to be good practice. This study results suggest that about 48.5% of the respondents changed their sanitary materials three times a day during menstruation, and this finding was also consistent with existing similar studies [7, 10]. Rechanging sanitary materials and regular washing of external genitalia maintaining proper hygiene are crucial for healthy menstrual management. One study suggests that those who washed the body and external genitalia with water only were 2.4 times more likely to be asymptomatic of one or more urogenital diseases than those who used both water and soap for washing [39]. This study shows that 33.7% of respondents washed their genitalia four or more times, and another 44.2% washed it three times per day. In comparison, 38.7% of adolescent girls used water and soap for washing their external genitalia. These findings were identical to two similar studies conducted in India [19, 40].

Disposal of sanitary materials properly is as important as other indicators of the menstrual hygiene management. Unhygienic disposal of sanitary materials in rivers, ponds, or even under the soil may increase the risk of infection of Hepatitis and HIV as sanitary materials soaked with the blood of an infected girl/woman may contain these Bacteria and viruses [38]. This study indicates that more than 55% of the participants disposed of their used sanitary materials under the soil. We found that 20.2% of adolescent girls threw their sanitary materials in ponds/rivers. This unhygienic behavior of disposing of used sanitary materials may put hundreds of people at risk of infection with fatal diseases like Hepatitis and HIV. Many people in Bangladesh use river and/or pond water for bathing and domestic washing purposes [41]. According to this study, the unmet need for disposable sanitary pads among non-pad users was 71.8%. Despite the high need, they (35.6%) could not use because of the high cost of sanitary pads and the embarrassment of buying pads from male shopkeepers (37.5%). Similar results were found in previous studies [1, 8, 37].

This study found that age, religion, family size, parental education, fathers’ income, perceived socioeconomic class, and age at first menstruation had a significant statistical association with menstrual hygiene management practices at the bivariate level, and these findings are similar to other studies [8, 25, 37, 42]. Being Muslims, members of a large family and children of less-educated fathers were associated with bad menstrual hygiene management practices. In Bangladesh, Muslim adolescent girls are more conservative, which may result in lower access to modern sanitary materials. On the other hand, girls from well-off families and daughters of educated parents may have more accessibility, affordability to sanitary pads, and more chance of getting information on menstrual hygiene management [8, 25, 37, 42, 43]. This study observed significant urban–rural differences in terms of menstrual hygiene management practices. Like all other indicators of demography and health [43, 44], the residents of urban areas have better menstrual hygiene management practices than rural areas. As a result, the percentage of respondents who did not participate in social activities, school, or work due to their last menstruation was significantly higher in rural areas than urban [43].

### Limitations of the study

This study used a cross-sectional research design. Thus, causal inferences about relationships observed in the data cannot be established. Another limitation of the study was that it was confined to a single geographic location (Rajshahi) in Bangladesh. Therefore, findings from the study may not represent the experiences of all adolescent girls in the country. Due to the sensitivity of the research topic, during the data collection procedure, we faced some challenges. One of the biggest challenges was the hesitancy of the heads of selected schools and the parents of primarily selected respondents' to allow their girls to participate in the survey. We had to go through a lengthy discussion to convince them in this regard. Even so, we faced complete refusal from at least one identified institution. Although we primarily intended to record responses by a trained interviewer, we had to rely on self-administration of the questionnaire by the respondents as the heads of the selected institutions did not allow male interviewers to conduct face-to-face interviews. Participating respondents were seated in a class environment where at least two or more respondents were seated on a single bench. We suspected that some of them had either been influenced by the fellow respondent or copied responses from others. A face-to-face interview would produce more quality data. We also noticed that some respondents were too shy to ask for help from their overseer even though they could not understand a particular question properly. Finally, the sample size of this study was relatively small; as a result, true population parameters may not be found.

## Conclusion

The primary objective of our study was to assess the menstrual hygiene management practices of adolescent girls, the urban–rural differences, if any, of the practices, and the determinants of such practices. Although the research findings show that many adolescent girls have information before reaching menarche about menstrual hygiene management, it should be considered that nearly one-fourth of respondents reach at menarche without any prior information about this. This study suggests that girls reaching menarche without prior information are more likely to practice bad menstrual hygiene management. Thus, measures must be taken to ensure information availability about menstrual management before a girl reaches her menarche through proper channels. As mothers and sister/s are the two significant sources of menstrual information, and as they often translate their misconception and attitude to adolescent girls, steps should be taken to change adult females’ attitudes and knowledge towards menstrual management. This study shows that more than one-fourth of adolescent girls use cloth, and another 35.2% sometimes use pads and sometimes cloths to manage their menses; therefore, measures must be taken to ensure a continuous supply of pads for all. Although nearly three-quarters of non-pad users wish to use sanitary pads, more than one-third cannot use them because of the high cost of sanitary pads. Among cloths users, more than one-fourth do not even want to use pads because it causes unnecessary money expending. Thus, it is essential to make pads available at affordable costs. Shyness to buy pads and unavailability of pads at nearby places also prevent many adolescent girls from pad use; thus, existing social norms regarding menstrual hygiene management must be changed. All those necessary measures must be taken to ensure adolescent girls’ healthy menstrual management to meet the SDG goals of good health and wellbeing, quality education, gender equality, and clean water and sanitation.

## Data Availability

This research was conducted as a part of partial fulfillment of the Bachelor of Social Sciences (BSS) honors degree from the Department of Population Sciences, University of Dhaka. The Department of Population Sciences, University of Dhaka has the right to reserve the data. Therefore, data cannot be provided in supplementary file or deposited in a public repository. However, the dataset could be obtained from the Department of Population Sciences, University of Dhaka (https://www.dpsdu.edu.bd/index.php/en/) or the corresponding author on reasonable request.

## Abbreviations

HPV: Human Papillomavirus
RTI: Reproductive tract infections
STD: Sexually transmitted disease
SDG: Sustainable Development Goal
